# Utilisation of sexual and reproductive health services among street children and young adults in Kampala, Uganda: does migration matter?

**DOI:** 10.1186/s12913-021-06173-1

**Published:** 2021-02-23

**Authors:** Mulekya F. Bwambale, Paul Bukuluki, Cheryl A. Moyer, Bart H. W. Van den Borne

**Affiliations:** 1 Department of Health Promotion and Education, Faculty of Health Medicine and Life Sciences, Maastricht University Care and Public Health Research Institute (CAPHRI), Maastricht, Netherlands; 2 Department of Social Work and Social Administration, School of Social Sciences, Makerere University College of Humanities and Social Sciences, Kampala, Uganda; 3 Departments of Learning Health Sciences and Obstetrics and Gynaecology, University of Michigan Medical School, Ann Arbor, USA

**Keywords:** Rural–urban migration, Street children and young adults, Sexual and reproductive health services, Uganda

## Abstract

**Background:**

While the nexus of migration and health outcomes is well acknowledged, the effect of rural–urban migration on the use of sexual and reproductive health (SRH) services has received less attention. We assessed the effect of rural–urban migration on the use of SRH services, while controlling for confounding, and whether there is a difference in the use of SRH services among migrant and non-migrant street children and young adults.

**Methods:**

Data were collected from 513 street children and young adults aged 12–24 years, using venue-based time-space sampling (VBTS). We performed multivariate logistic regression analysis using Stata 16.0 to identify factors associated with SRH services use, with rural–urban migration status as the main predictor. Participants were further classified as new migrants (≤ 2 years of stay in city), established migrants (> 2 years of stay in city) or non-migrants (lifelong native street children) with no rural–urban migration history.

**Results:**

Overall, 18.13% of the street children and young adults had used contraception/family planning, 58.67% had tested for human immunodeficiency virus (HIV) and knew their status and 34.70% had been screened for sexually transmitted infections (STIs). Non-migrants were 2.70 times more likely to use SRH services (HIV testing, STI screening and family planning) compared to the migrants (aOR = 2.70, 95% CI 1.23–5.97). Other factors associated with SRH services use among street children and young adults include age (aOR = 4.70, 95% CI 2.87–7.68), schooling status (aOR = 0.33, 95% CI 0.15–0.76), knowledge of place of care (aOR = 2.71, 95% CI 1.64–4.46) and access to SRH information (aOR = 3.23, 95% CI 2.00–5.24).

**Conclusions:**

SRH services utilisation among migrant street children and young adults is low compared to their non-migrant counterparts and is independently associated with migration status, age, schooling status, knowledge of place of care and access to SRH information. Our findings call for the need to design and implement multi-dimensional interventions to increase the use of SRH services among street children and young adults, while taking into consideration their migration patterns.

**Supplementary Information:**

The online version contains supplementary material available at 10.1186/s12913-021-06173-1.

## Background

Sexual and reproductive health (SRH) is an essential part of universal health coverage and is primarily incorporated into the United Nations’ Sustainable Development Goal 3 on healthy lives and well-being, which ensures universal access to SRH services, including family planning, information and education [1]. Young people aged 10–24 years lie at the very heart of sustainable development as they are the ones most affected by poverty, conflict and migration [2]. Young people have the agility and a greater ability to adopt and maintain safe behaviours, alter the situation against sexually transmitted infections (STIs), human immunodeficiency virus (HIV) and early pregnancy than adults do [3]. Nonetheless, issues of adolescents’ access to quality and confidential SRH services are often disregarded when drafting policies [4, 5].

The need to address the SRH rights of adolescents and young people is critical in improving their SRH outcomes [6]. However, lack of awareness of SRH information and where to seek care as well as maltreatment by health workers often limit young people’s access to SRH services [7]. Young women, in particular, face numerous challenges that prevent them from using quality SRH services, including stigma, fear of contraceptive side effects and unaffordable healthcare costs [8, 9]. Within the context of urban health, street children and young adults face several health risks, including high rates of unwanted pregnancies, STIs, sexual violence and poor maternal and child health outcomes [10, 11].

The topic of rural–urban migration has been at the centre of debates about urbanisation and development at the global, continental and national levels due to its influence on social and economic underdevelopment of rural areas [12, 13]. While the migration process is acknowledged a social determinant of health [14], literature on the effect of internal migration on the SRH services use is limited, with most studies exploring the nexus of international migration and maternal health outcomes conducted in high-income countries [15, 16]. For instance, in Italy, female immigrants were found to be at greater risk of receiving inadequate healthcare services compared to the native females [17]. A few studies in sub-Saharan Africa on the impact of migration on healthcare indicate that migrants exhibit a disproportionately higher risk of contracting HIV and other STIs compared to non-migrants [18, 19].

In Uganda, an increasing internal migration phenomenon resulting from urbanisation has caused an influx of street children and young people who are most at risk in all fronts [20]. All regions of Uganda have experienced rural–urban migration with most street children and youth reported to be migrating from the Karamoja subregion, Eastern Uganda [21]. Despite efforts to improve the policy space around adolescent SRH [22], implementation of SRH programmes – especially those impacting the most vulnerable urban groups, such as street children and young adults – remains problematic. The lack of recognition of street children and young adults in policies and strategic plans of the Kampala Capital City Authority (KCCA) [23], may lead to social exclusion of this already marginalised group from accessing basic social services. Moreover, the schism between policy and practice – those making policies about migrants’ entitlements versus those providing health services to migrants – remains apparent.

The question of whether rural–urban migration influences SRH services utilisation among street children and young adults in urban environments in Uganda is not fully understood. Our study attempts to fill this knowledge gap by investigating the factors that influence the use of SRH services among street children and young adults in Kampala city, with rural–urban migration status as the main predictor of interest. A strategic focus on the nexus of rural–urban migration and access to quality SRH services among street children and young adults is critical in designing future interventions to improve the SRH of urban poor street children and youth. This is guided by the United Nations’ 2030 Agenda for the Sustainable Development Goals (SDGs) principle of ‘leave no one behind’ and the KCCA’s aspiration for improved quality of life and human capital needed to support city economic development [23, 24].

### Conceptual framework

The conceptual framework of this study largely borrows from the social ecological model (SEM) of health behaviour to understand the interaction between several predictors of SRH services use among street children and young adults, including rural–urban migration status [25, 26]. Ecological models explicitly consider the individual, interpersonal, broader community, organisational and policy contexts of behaviour, while incorporating social influences, which in our study context, was deemed most appropriate [27, 28]. Within the context of migration, other competing theories may explain the association between migration and reproductive health behaviour. These include the healthy migrant effect model which posits that, over time, individuals who migrate could have healthier behaviours and better outcomes than the native population [29]. We applied the social ecological model to guide the interviews, data analysis and interpretation of the findings.

## Methods

Using a cross-sectional study design, we assessed the association of rural–urban migration with SRH services utilisation, while controlling for socio-demographic factors. We also determined whether differences in the use of SRH services exist among migrant and non-migrant street children and young adults in Kampala, Uganda. The study was implemented in three of the five divisions of Kampala capital city, namely the Makindye, Central and Rubaga divisions. Kampala capital city comprises a night population of approximately 1.50 million people. Availability of health facilities in Kampala capital city remains the highest in the country at 8.40 facilities per 10,000 population [30].

We conducted face-to-face interviews among a random sample of 513 children and young adults aged 12–24 years who a) had continuously stayed in Kampala capital city for at least 3 months prior to the study, b) spent most of their time on the streets of Kampala capital city, and c) consented to participate in the study. The study applied venue-based time-space sampling (VBTS) technique. VBTS techniques are widely applied in contexts where no comprehensive lists or census data of the target population exist, and to sample hard-to-reach populations using peer-driven recruitment [31]. Venues were defined as public or private locations within the city where street children and young adults congregate, live and spend most of their daytime to earn a livelihood. These included urban spaces, such as streets, bars and restaurants; markets; parks; bus stations; traffic lights and road junctions, as well as temporary shelters.

First, to identify the parishes and geographic venues where street children and youth congregate during the day and could be contacted, we conducted a pre-survey rapid mapping exercise that involved interviews with key stakeholders (urban local leaders and service providers) and geo-mapping of parishes and estimated populations of street children at each venue. In the absence of any mapping or longitudinal data of locations of street children, we considered this pre-survey mapping exercise as the most feasible and appealing approach to inform our sampling strategy. The parishes served as the primary sampling units for data collection and were selected with equal probability. Therefore, parishes with more congregation venues had proportionately a high sample of street children interviewed. New venues were added if the research assistant noticed other locations in the field that were not on the initial list. The identification of the venues, street children and young adults was made possible with the help of the local urban leaders, service provider non-government organisations (NGOs) and street children’s landlords or caregivers. Overall, we enlisted a total of 167 venues in 27 parishes from the three selected divisions.

Second, at each of the selected venues, we applied respondent-driven sampling (RDS) to recruit eligible street children and young adults using their network groups to participate in the interviews. Since the number of street children and young adults during the fixed time interval was small (15 or fewer), we interviewed all eligible participants found in each venue. RDS is a chain referral sampling method that produces a stable sample regardless of the make-up of initial recruits [32].

### Dependent variables

We created a binary composite variable ‘SRH services utilisation’ from three dependent variables, namely a) ever used contraception or family planning in lifetime, b) HIV testing in the past 12 months, and c) history of STI screening in the 12 months preceding the survey. Hence, a participant who responded ‘yes’ to the three questions was considered to have used SRH services:

- *In the past 12 months, have you sought for STI testing/screening? (Yes/No);*
- *In the last 12 months, have you taken an HIV test and know your HIV status? (Yes/No); and*
- *Have you ever used any modern family planning? (Yes/No).*

A positive (‘yes’) response to any one of these questions was regarded as SRH services utilisation. Positive responses to the above questions were further validated with follow-up questions on the type of family planning (FP) methods used. Previous studies have used similar categorisation of SRH services and self-reports as a measure of health service utilisation and is thus deemed appropriate for our study [33, 34]. Questions to measure SRH services utilisation were adapted from the Demographic and Health Surveys (DHS) Program Questionnaire [35].

### Independent variables

Rural–urban migration aspects measured comprised place (district/region) of origin, duration of stay, intra-urban mobility and number of return movements to the city. Specific questions included:

- *In which district of Uganda were you born (where your family/parents live)?*
- *In which year and month did you first come to settle in Kampala capital city?*
- *For how long you have stayed or lived in Kampala capital city?*
- *How many places have you stayed in or moved since you first settled/arrived in the city?*
- *In the past 24 months, have you returned to your district/region of origin/birth? (Yes/No). If yes, how many times did you go back to your district of birth/origin and returned to the city (round trips)?*

In this study, street children and young adults with a rural–urban migration experience were classified as ‘migrants’, while street children and young adults with no rural–urban migration history were considered ‘non-migrants’. Individuals were further classified as new migrants (≤ 2 years of stay in city), established migrants (> 2 years of stay in city) or non-migrants (lifelong native) street children and young adults with no rural–urban migration history. Previous studies have used similar categorisations for migration status [36, 37]. Street children and young adults who had more than one repeat movement to their district of origin in the 24 months preceding the survey were classified as ‘circular migrants’. Other independent variables included participant’s characteristics such as age, sex, marital status, schooling experience, education level, daily income earned and living arrangements, as illustrated in the study framework (Fig. 1). Since existing surveys such as Demographic Health Surveys (DHS) and Multiple Indicator Cluster Surveys (MICS) do not collect data on migration, questions on the migration module were developed primarily for this study (Additional file 1).

### Procedures

Between May and July 2019, data were collected using a pretested interviewer-administered semi-structured instrument programmed using SurveyCTO on android mobile tablets in English and two local languages, *Luganda* and *Ngakarimajong*, which are commonly spoken among street children and young adults. The electronic tool ensured minimisation of errors and completeness of the interviews. Since street children and young adults are connected through social networks, eligible respondents were recruited through respondent-driven sampling in which street children and young adults had to lead the researcher to their peers for inclusion in the study. To ensure comprehension and a high response rate among the study participants, a team of 14 trained male and female research assistants conducted the interviews. All interviews took place in private spaces at the selected venues and along the streets. Participants who are unable to provide the required information due to illness were excluded. Written consent from the street children and young adults was obtained before interviews were conducted. Informed written consent was obtained from caregivers of children younger than 14 years, while study participants aged 14 years and above consented on their own. The National Council of Science and Technology (NCST) guidelines on ethics and research with human subjects considers the age group 14–17 years to be emancipated minors and hence able to provide own consent without parental or guardian permission [38].

### Data analysis

To examine the factors associated with SRH services utilisation, including rural–urban migration status as the main explanatory variable of interest, we performed a stepwise binary logistic regression analysis using Stata version 16.0, while controlling for confounding. Stepwise binary logistic regression has been applied elsewhere [39]. The choice of variables in the final multivariate regression analysis models was guided by the conceptual framework while assessing for multicollinearity among the predictor variables. We first tested for the independent effects of each outcome variable (HIV testing, STI screening and FP use) using separate regression models, followed by the combined regression model (SRH service utilisation). We examined possible differences between the models by using the same explanatory variables across all the models and R-squared values. Also, we performed a sub-analysis of factors associated with SRH services utilisation, stratified by migration status. All analyses were two-tailed and a *p*-value of 0.05 or less was deemed statistically significant. Lastly, we report results from the four multivariate logistic regression analysis models.

## Results

### Background characteristics

Table 1 shows the background characteristics of street children and young adults by migration status. Overall, 82.85% (*n* = 425) and 17.15% (*n* = 88) of street children and young adults were migrant and non-migrant, respectively. Most of the street children and young adults were aged 18 years and above and most were male. More migrants (76.50%) than non-migrants (58.57%) had attained pre-school or primary school education while the majority (56.82%) of non-migrants had attained secondary education. Almost nine out of ten street children and young adults were not in school. Most (79.53%) of the street children and young adults were not married and only 16.76% were either married or living with a partner. In terms of living arrangement, most (63.38%) of the new migrants stayed with a sexual partner or friend compared to established migrants (57.45%) and non-migrants (37.50%). Non-migrant street children and young adults earned more than the threshold value of one USD daily compared to new and established migrants. About half of the street children and young adults were orphans. There was no significant difference in orphanhood status by migration status.

### Prevalence of SRH services utilisation

Table 2 shows a crosstabulation of SRH services utilisation among street children and young adults by migration status. Overall, 82.85% and 17.15% of street children and young adults were migrant and non-migrant, respectively. Less than two thirds (61.99%) of the street children and young adults received at least one component of the SRH services (HIV testing, STI screening and FP use) in the last 12 months. Across all the components, SRH services utilisation was generally lower among the migrants compared to non-migrant street children and young adults. More (30.68%) of the non-migrant street children and young adults had used a modern family planning method compared to established migrants (17.73%) and new migrants (14.44%). About three quarters (73.86%) of the lifelong native street children and young adults had tested for HIV in the past 12 months and knew their status compared to 51.76 and 63.12% of new migrants and established migrants, respectively.

Similarly, the prevalence of STI screening in the past 12 months was low among migrant street children and young adults, with 26.41% of new migrant and 38.30% of established migrants screened for STIs compared to 55.68% of their non-migrant counterparts. The differences in SRH services utilisation are significant at the 0.05 *p*-value. Additional findings (not shown in the table) revealed a condom as the most commonly used FP method (77.03%). The majority (68.39%) of the street children and young adults reported accessing SRH services through public sector primary health facilities compared to 31.61% access from non-governmental organisations (NGO) and private health facilities.

### Factors associated with SRH services utilisation

Table 3 shows findings from the bivariate logistic regression analysis of socio-demographic and migration variables on utilisation of SRH services (HIV testing, STI screening and FP use) among street children and young adults. SRH services utilisation was associated with increasing age, with those aged above 18 years being 5.80 times more likely to use SRH services than the younger age group (cOR = 5.84, 95% CI 3.95–8.64). Regarding marital status, street young adults in conjugal relationships were 2.50 times more likely to use SRH services than street young adults in non-conjugal relationships (cOR = 2.47, 95% CI 1.43–4.26). Individuals who perceived themselves as permanent residents of the city were twice as likely to use SRH services than the seasonal street children (cOR = 1.93, 95% CI 1.32–2.81). Utilisation of SRH services among street children and young adults was more than doubled with attainment of post primary education (cOR = 2.71, 95% CI 1.69–4.34). Intra-urban mobility (movement between urban spaces) was associated with SRH services use among street children and young adults (cOR = 2.13, 95% CI 1.43–3.15). However, the odds of using SRH services decreased with migration status (cOR = 0.52, 95% CI 0.36–0.74), duration of stay (cOR = 0.64, 95% CI 0.42–0.97) and ethnicity (cOR = 0.56, 95% CI 0.37–0.82). The reduced odds of using SRH services further supports our hypothesis that migration inhibits the use SRH services among migrant street children and young adults in urban spaces.

### Migration as the main predictor of SRH services utilisation

Table 4 shows findings from the four multivariate binary logistic regression models with SRH services utilisation (model 1), STI screening (model 2), HIV testing (model 3) and use of family planning (model 4) as the main outcome variables. The final models included ten predictors of SRH services utilisation, including rural–urban migration status, age, sex, daily income, schooling status and other known predictors of SRH services utilisation. The findings reveal that non-migrant street children and young adults were 2.70 times more likely to utilise SRH services than their migrant counterparts (aOR = 2.71, 95% CI 1.23–5.97), implying that SRH services utilisation is generally low among new and established migrants compared to non-migrants or lifelong native street children and young adults. We did not find a significant relationship between circular and non-circular migrants (street children and young adults who had more than one repeat movement between the city and district of origin) and the use of SRH services.

### Other predictors of SRH services utilisation

Other factors that predicted the utilisation of SRH services among street children and young adults include age, knowledge of place of care for FP services and access to SRH information. Street young adults aged 18–24 years were 4.70 times more likely to utilise SRH services than those aged 12–17 years (aOR = 4.70, 95% CI 2.87–7.68). The odds of having used modern FP methods was 67.00% lower if street children and young adults were in school as opposed to their out-of-school counterparts (aOR = 0.33; 95% CI 0.15–0.76). Street children and young adults with knowledge of a place of SRH care were 3.20 times more likely to utilise FP services compared to individuals without knowledge of a place of SRH care (aOR = 3.37, 95% CI 2.04–5.34). Street children and young adults who reported having received SRH education in the past 6 months were 2.70 times more likely to use SRH services than those without SRH education (aOR = 2.71, 95% CI 1.64–4.46).

Results from the multivariate regression models 2, 3 and 4 revealed findings similar to those of the pooled outcome (SRH services use) with some slight variations in the measures of the strength of association. STI screening and HIV testing services use were associated with migration status, age and SRH education. For instance, the association between migration status and SRH services utilisation was insignificant except for FP services in which there was a 70.00% lower likelihood of having used FP services among Christians than non-Christians (aOR = 0.30, 95% CI 0.15–0.62). Street children and young adults aged 18–24 years were five times more likely to use FP services than their counterparts aged 12–18 years (aOR = 5.30, 95% CI 2.28–12.33). The odds of having ever used FP was 10.44 times higher if street children and young adults knew the place of FP care as opposed to not knowing the place of FP care (aOR = 10.44, 95% CI 4.87–22.38). We did not find any significant difference in use of FP services between new migrants, established migrants and non-migrant (lifelong native) street children and young adults (aOR = 0.85, 95% 0.37–1.92).

Multivariate regression analysis based on sample stratification by migration status revealed significant differences in the use of SRH services among street children and young adults with regards to age, schooling status, SRH education and place of FP care. Among new migrants alone, increased SRH services utilisation was associated with age (aOR = 6.32, 95% CI 3.28–12.17), SRH education in the past 6 months (aOR = 2.48, 95% CI 1.32–4.65) and knowing a place of FP care (aOR = 2.68, 95% CI 1.44–4.99). Among established migrants, the odds of using SRH services increased 4-fold if street children were older than 18 years (aOR = 3.69, 95% CI 1.27–10.72), received SRH education (aOR = 3.62, 95% CI 1.19–10.99) or knew the place of FP care (aOR = 3.54, 95% CI 1.34–9.30). Similarly, among non-migrants, the odds of SRH services utilisation were increased with age (aOR = 8.76, 95% CI 1.38–55.52). We found no difference in SRH services utilisation between sexes.

## Discussion

This is the first study in Uganda that primarily investigates the nexus of rural–urban migration and SRH services utilisation among street children and young adults using quantitative methods, through the social ecological lens. Our study reveals rural–urban migration as a key determinant of SRH services utilisation among street children and young adults in Kampala, with migrants being disproportionately affected compared to non-migrants. Other predictors of SRH services utilisation among street children and young adults included age, schooling status, access to SRH education and knowledge of a place of FP care. Religion was a major predictor of the use of contraception.

Returning to the question of whether rural–urban migration influences SRH services utilisation, the low use of SRH services (HIV testing, STI screening and FP) among migrants compared to non-migrants confirms our hypothesis that non-migrant street children and young adults are likely to make better use of SRH services than rural–urban migrants. It also demonstrates migration status as a barrier to access to and use of SRH services (HIV testing, STI screening and FP) among rural–urban migrant street children and young adults living in urban environments. This finding is plausible given the social context within which migrant street children and young adults live, adapt and socialise while on the streets of Kampala.

Conversely, the odds of using SRH services were 1.40 times higher among established migrants (> 2 years of stay in city) compared to new migrants (≤ 2 years of stay in city). It should be noted that street life is a process that requires adaptation to the new street environment as the newcomers may take some time to establish social and peer support networks, which are critical for healthcare support. As such, street children and young adults, especially established migrants, must duly navigate the challenges of the streets while also adapting to the new street culture and language [40, 41].

Reduced utilisation of family planning, especially among rural–urban migrant street young adults could potentially result into increased fertility that could further escalate the country’s annual population growth rate of 2.88%, which seems to be growing faster than government’s capacity to deliver vital services [42]. However, this result contrasts sharply with the Kenyan study in which use of modern contraception was higher in migrants than non-migrant women [43]. The difference in our findings could be attributed to different methods and study populations used. Unlike our study, the Kenyan study utilised national demographic and health survey data of sexually active women of reproductive age group and looked at different migration streams to explain the differences.

In our study, we observed an association between age and SRH services utilisation. This finding may suggest that street young adults are stronger and therefore can navigate the complex urban healthcare system with ease compared to their young counterparts, who may require support from adults in seeking healthcare services. It is possible that street young adults are involved in health-compromising practices such as drugs and risky sexual behaviour and are therefore more likely to seek healthcare than their younger counterparts. The reduced odds of using SRH services, especially HIV testing services, among in-school street children and young adults as opposed to their out-of-school counterparts may suggest limited access to SRH information and services within the school environment due to restrictive policies.

In Uganda, the current policy does not allow distribution of SRH commodities, thus prohibiting the sexually active learners within from accessing them within the school environment. Such restrictive educational policies that include sanctions against young people found to be in possession of condoms while in school hinder effective implementation of sexual behaviour interventions in many low- to middle-income countries [44]. When in school, street children may be omitted from SRH services that are provided to their out-of-school counterparts within urban community environment. Within the local context, with most street children being out-of-school, the community environment remains the most appropriate avenue for delivery of SRH information and services to this marginalised group of poor urban youth. Earlier studies have demonstrated the role of access to SRH information in contraceptive use among young people [45].

In our study, religion strongly influenced the uptake of family planning among street children and young adults. The low uptake of family planning among Christians compared to non-Christians could be attributed to the myths, misconceptions, cultural and religious beliefs about modern contraception which are widely held by many rural communities in Uganda from which the street children and young adults originate. Previous qualitative studies done in Uganda and Tanzania confirm that religious and cultural beliefs remain an impediment to uptake of family planning methods among women and men of reproductive age [46, 47]. This result may suggest a high unmet need for contraception among female street young adults and provides an opportunity for responders to engage urban street children and young adults on changing their religious perceptions and practices on modern contraceptives. Since young people may be ardent followers of their faith leaders, involving the street children and young adults is critical in tackling their SRH rights and needs.

The absence of an association between circular migration and SRH services utilisation could be attributed to limited opportunities to access to SRH and other support services among street children and young adults during the migration cycle. It may imply that characteristics of street children and young adults who have stayed for long periods (spent more than 2 years in the city) without going back home may not differ from those who had repeated movements between the city and districts of origin in the past 2 years. The implications of rural–urban circular migration on access to SRH services among urban street children and young adults may require further exploration.

This study had some strengths and limitations. First, we were able to establish the association between rural–urban migration and SRH services utilisation among street children and young adults as the main explanatory variable, while controlling for confounding as guided by the study theory. Second, the use of a large sample size with adequate power to detect the minimum meaningful difference in establishing a relationship between predictors and SRH services utilisation is another strength. Third, it is possible that some individuals who were categorised as migrants in the study used contraception before migrating to the city and are hence likely to increase the disparity in the utilisation of SRH services among migrants and non-migrants. Fourth, our data are cross sectional and therefore preclude our ability to determine the direction of causality. Fifth, we did not control for sexual behaviour and participants’ household characteristics, which might possibly confound the relationship between migration and SRH services use. Sixth, the absence of a well-defined formal housing structure for street children and young adults in the urban spaces limits our analysis of household characteristics. Since our study sample was representative of street children and young adults from across all regions of Uganda, our study findings could be generalisable to all urban street children and young adults.

Regarding the study’s theoretical underpinning, our study findings illustrate the synergetic relationship between individuals, interpersonal and their social environment factors which interact to influence the use of SRH services among street children and young adults through the social ecological lens. Previous research on SRH services utilisation has adopted the socio-ecological model [48]. The observed low utilisation of SRH services among new and established migrants compared to non-migrant (lifelong native) street children and young adults contrasts with the healthy migrant effect model which posits that, over time, individuals who migrate could have healthier behaviours than the native population [29].

Finally, this study provides new knowledge on the understanding of the nexus of rural–urban migration and SRH services utilisation among street children and young adults in Kampala, Uganda. It highlights the need for future research on the impact of rural–urban migration on street children and young adults’ SRH behaviour and outcomes.

## Conclusions

In conclusion, utilisation of SRH services among street children and young adults in Kampala is generally low and is independently associated with rural–urban migration status, age, schooling status, knowledge of place of SRH care and access to SRH information. Rural–urban migrant street children and young adults are less likely to utilise SRH services than their non-migrant counterparts. Our study findings point to the need for the Kampala Capital City Authority (KCCA) and key responders to design multi-level effective interventions, guided by the socio-ecological lens, to improve equitable access to and use of SRH services and information among street children and young adults in the Kampala metropolitan area and ultimately contribute to their improved SRH.

Multi-level effective interventions at individual, community and societal levels should include delivery of integrated SRH and HIV outreach and mobile clinic services to the urban spaces where street children and young adults live or congregate during the daytime; tailored SRH and HIV peer-to-peer education messages using multimedia campaigns; and delivery of street child-responsive SRH services at Kampala city’s healthcare facilities. At the society level, improving the policy environment and living conditions of street children and young adults, especially the rural–urban migrants, is critical in achieving universal access to adolescent SRH services for all. This will require the recognition of street children and young adults as a priority vulnerable group in national and urban policies and plans.

## Supplementary Information

**Additional file 1.** Semi-Structured Questionnaire – Migration Module.

**Additional file 2: Figure 1**. A socio-ecological framework showing independent factors associated with use of SRH services among street children and young adults, Kampala, Uganda, 2019. **Table 1.** Background characteristics of street children and young adults by migration status, Kampala, Uganda, 2019. **Table 2.** Crosstabulation of SRHR services utilisation among street children and young adults by migration status, Kampala, Uganda, 2019. **Table 3.** Bivariate analysis of selected predictors and migration aspects with SRH services utilisation among street children and young adults in Kampala, Uganda, 2019. **Table 4.** Multivariate analysis of demographic and migration status with SRH services utilisation among street children and young adults in Kampala, Uganda, 2019.

## Data Availability

The dataset used and/or analysed during the current study is available from the corresponding author on reasonable request.

## Abbreviations

aOR: Adjusted odds ratio
cOR: Crude odds ratio
FP: Family planning
HIV: Human immunodeficiency virus
RDS: Respondent-driven sampling
SRH: Sexual and reproductive health
STIs: Sexually transmitted infections
VBTS: Venue-based time-space sampling
