# Supplementary material for: Utilisation of sexual and reproductive health services among street children and young adults in Kampala, Uganda: does migration matter?
Source: BMC Health Serv Res. 2021 Feb 23;21:169. doi: 10.1186/s12913-021-06173-1 (PMC7903651; doi:10.1186/s12913-021-06173-1)
Supplement: Supplementary file 1 — Additional file 1. Semi-Structured Questionnaire – Migration Module. [file 12913_2021_6173_MOESM1_ESM.docx]

**Additional File 1: Semi-Structured Questionnaire – Migration Module**

**Instructions:** *As we come to end of the interview, I would like to ask you a few questions regarding your movements within, to and from Kampala city.*

1. In which district of Uganda were you born? (where your family/parents live)? 1. Kampala 2. Other district, specify _______ (if Kampala, skip to Q7)
2. In which **year and month** did you first come to settle/arrive in Kampala city? Year ____ Month _____
3. For how long have you stayed in Kampala continuously without going back to your district of birth/origin? ______ (record in months)
4. How many places have you stayed in or moved since you first settled/arrived in the city? _____
5. In the past 24 months, have you returned to your district/region of birth/origin? 1.Yes 2.No, If No, skip to 7.
6. If yes to Q5, how many times did you go back to your district of birth/origin and returned to the city (round trips)? _____
7. Do you have any plans of moving to settle in another town/city in Uganda? 1.Yes 2.No,

Thank you for taking the time to talk to us!!
