# Supplementary material for: Utilisation of sexual and reproductive health services among street children and young adults in Kampala, Uganda: does migration matter?
Source: BMC Health Serv Res. 2021 Feb 23;21:169. doi: 10.1186/s12913-021-06173-1 (PMC7903651; doi:10.1186/s12913-021-06173-1)
Supplement: Supplementary file 2 — Additional file 2: Figure 1. A socio-ecological framework showing independent factors associated with use of SRH services among street children and young adults, Kampala, Uganda, 2019. Table 1. Background characteristics of street children and young adults by migration status, Kampala, Uganda, 2019. Table 2. Crosstabulation of SRHR services utilisation among street children and young adults by migration status, Kampala, Uganda, 2019. Table 3. Bivariate analysis of selected predictors and migration aspects with SRH services utilisation among street children and young adults in Kampala, Uganda, 2019. Table 4. Multivariate analysis of demographic and migration status with SRH services utilisation among street children and young adults in Kampala, Uganda, 2019. [file 12913_2021_6173_MOESM2_ESM.docx]

**Figure and Tables**

**Figure 1 A socio-ecological framework showing independent factors associated with use of SRH services among street children and young adults, Kampala, Uganda, 2019.**

**Table 1: Background characteristics of street children and young adults by migration status, Kampala, Uganda, 2019**

| **Characteristic** | **New migrants (%)**  **(n=284)** | **Established migrants (%)**  **(n=141)** | **Non-migrants (%)**  **(n=88)** | **Total**  **(%)**  **(N=513)** |
| --- | --- | --- | --- | --- |
| Age (in complete years) ** |  |  |  |  |
| 12–17 | 44.72 | 26.24 | 35.23 | 38.01 |
| 18–24 | 55.28 | 73.76 | 64.77 | 61.99 |
|  |  |  |  |  |
| Mean age (SD) | 17.94 (3.04) | 19.11 (2.98) | 19.08 (3.43) | 18.46 (3.14) |
|  |  |  |  |  |
| Sex ** |  |  |  |  |
| Male | 47.89 | 66.67 | 82.95 | 59.06 |
| Female | 52.11 | 33.33 | 17.05 | 40.94 |
| Schooling status** |  |  |  |  |
| Out of school | 93.66 | 87.23 | 80.68 | 89.67 |
| In school | 6.34 | 12.77 | 19.32 | 10.33 |
|  |  |  |  |  |
| Highest education attained ** |  |  |  |  |
| Primary | 76.50 | 72.13 | 39.77 | 67.68 |
| Secondary | 23.04 | 27.05 | 56.82 | 31.15 |
| Tertiary | 0.46 | 0.82 | 3.41 | 1.17 |
| Marital Status ** |  |  |  |  |
| Married/cohabiting | 21.48 | 14.18 | 5.68 | 16.76 |
| Not married/single | 77.11 | 80.85 | 85.23 | 79.53 |
| Divorced/separated/widowed | 1.41 | 4.96 | 9.09 | 3.71 |
|  |  |  |  |  |
| Living arrangement ** |  |  |  |  |
| Stays alone | 11.97 | 12.06 | 12.50 | 12.09 |
| Stays with partner/friends | 63.38 | 57.45 | 37.50 | 57.30 |
| Stays with parents | 3.52 | 12.06 | 32.95 | 10.92 |
| Stays with siblings/other | 21.13 | 18.44 | 17.05 | 19.69 |
|  |  |  |  |  |
| Daily income earned (USD)** |  |  |  |  |
| <1 USD | 40.47 | 21.67 | 19.05 | 32.27 |
| ≥1 USD | 59.53 | 78.33 | 80.95 | 67.73 |
| Orphanhood status |  |  |  |  |
| Orphaned | 46.83 | 53.90 | 53.41 | 49.90 |
| Non-orphaned | 53.17 | 46.10 | 46.59 | 50.10 |
|  |  |  |  |  |

*** Significant at the 0.05 p-value.*

**Table 2: Crosstabulation of SRHR services utilisation among street children and young adults by migration status, Kampala, Uganda, 2019**

| **Characteristic** | **New migrants**  **% (n)** | **Established migrants**  **% (n)** | **Non-migrants**  **% (n)** | **Total**  **% (n)** |
| --- | --- | --- | --- | --- |
| Tested for HIV & knows status in the past 12 months** |  |  |  |  |
| No | 48.24(137) | 36.88(52) | 26.14(23) | 41.33 (212) |
| Yes | 51.76(147) | 63.12(89) | 73.86(65) | 58.67 (301) |
| Used modern family planning method** |  |  |  |  |
| No | 85.56 (243) | 82.27 (116) | 69.32 (61) | 81.87 (420) |
| Yes | 14.44 (41) | 17.73 (25) | 30.68 (27) | 18.13 (93) |
| Screened for STI in the past 12 months ** |  |  |  |  |
| No | 73.59 (209) | 61.70 (87) | 44.32 (39) | 65.30 (335) |
| Yes | 26.41 (75) | 38.30 (54) | 55.68 (49) | 34.70 (178) |
| Used SRH services (HIV test, STI screening or FP) ** |  |  |  |  |
| No | 44.72 (127) | 34.04 (48) | 22.73 (20) | 38.01 (195) |
| Yes | 55.28 (157) | 65.96 (93) | 77.27 (68) | 61.99 (318) |
| Knows place of care for FP services ** |  |  |  |  |
| No | 55.63 (158) | 52.48 (74) | 28.41 (25) | 50.10 (257) |
| Yes | 44.37 (126) | 47.52 (67) | 71.59 (63) | 49.90 (256) |

*** Significant at the 0.05 p-value*

**Table 3:** **Bivariate analysis of selected predictors and migration aspects with SRH services utilisation among street children and young adults in Kampala, Uganda, 2019**

| **Characteristic** | **Utilisation of SRH services** | | **cOR (95% CI)** |
| --- | --- | --- | --- |
|  | **Never used**  **n (%)** | **Ever used**  **n (%)** |  |
| **Sex (N=513)** |  |  |  |
| Male | 110 (56.41) | 193 (60.69) | 1 |
| Female | 85 (43.59) | 125 (39.31) | 0.84 (0.58–1.20) |
| **Age in years **** |  |  |  |
| 12–17 | 123 (63.08) | 72 (22.64) | 1 |
| 18–24 | 72 (36.92) | 246 (77.36) | 5.84 (3.95–8.64) |
| **Marital Status **** |  |  |  |
| Not married | 176 (90.26) | 251(78.93) | 1 |
| Married/cohabiting | 19 (9.74) | 67 (21.07) | 2.47 (1.43–4.26) |
| **Perceived residence status **** |  |  |  |
| Mobile/seasonal | 137 (70.26) | 175 (55.03) | 1 |
| Permanent | 58 (29.74) | 143 (44.97) | 1.93 (1.32–2.81) |
| **Highest education attained**** |  |  |  |
| Primary | 121 (80.67) | 168 (60.65) | 1 |
| Secondary +Tertiary | 29 (19.33) | 109 (39.35) | 2.71 (1.69–4.34) |
| **Schooling status **** |  |  |  |
| Out of school | 164 (84.10) | 296 (93.08) | 1 |
| In school | 31 (15.90) | 22 (6.92) | 0.39(0.22–0.70) |
| **Daily income earned**** |  |  |  |
| Less than 1 USD | 65 (38.92) | 77 (28.21) | 1 |
| 1 USD and above | 102 (61.08) | 196 (71.79) | 1.62 (1.08–2.44) |
| **Migration status** |  |  |  |
| New migrants (≤ 2 years of stay) | 127 (65.13) | 157 (49.37) | 1 |
| Established migrants (> 2 years of stay) | 48 (24.62) | 93 (29.25) | 1.57 (1.03–2.38) |
| Non migrants (lifelong native street children) | 20 (10.26) | 68 (21.38) | 2.75 (1.59–4.78) |
| **Intra-urban mobility **** |  |  |  |
| 1 place (move) | 99 (56.57) | 95 (38.00) | 1 |
| 2 or more places (multiple moves) | 76 (43.43) | 155 (62.00) | 2.13 (1.43–3.15) |
| **Place of origin/birth**** |  |  |  |
| Outside Kampala district | 175 (89.74) | 250 (78.62) | 1 |
| Kampala district | 20 (10.26) | 68 (21.38) | 2.38 (1.48–4.06) |
| **Region of birth**** |  |  |  |
| Other regions | 126 (64.62) | 171 (53.77) | 1 |
| Central region | 68 (35.38) | 147 (46.23) | 1.57 (1.09–2.27) |
|  |  |  |  |
| **District of birth/origin**** |  |  |  |
| Born outside Kampala | 175 (89.74) | 250 (78.62) | 1 |
| Born in Kampala | 20 (10.26) | 68 (21.38) | 2.38 (1.39–4.02) |
|  |  |  |  |
| **Ethnicity (tribe)**** |  |  |  |
| Other tribes | 113 (57.95) | 226 (71.07) | 1 |
| Ngakarimajong | 82 (42.05) | 92 (28.93) | 0.56 (0.37–0.82) |
|  |  |  |  |
| **Circular movement** |  |  |  |
| Non-circular migrant | 94 (74.02) | 112 (71.34) | 1 |
| Circular migrant | 33 (25.98) | 45 (28.66) | 1.14 (0.68–1.94) |

*** Significant at the 0.05 p-value.*

**Table 4:** **Multivariate analysis of demographic and migration status with SRH services utilisation among street children and young adults in Kampala, Uganda, 2019**

| **Predicator variable** | **Model 1**  **(SRH use)**  **aOR (95% CI)** | **Model 2**  **(STI screening)**  **aOR (95% CI)** | **Model 3**  **(HIV testing)**  **aOR (95% CI)** | **Model 4**  **(Ever used FP)**  **aOR (95% CI)** |
| --- | --- | --- | --- | --- |
| Migration status |  |  |  |  |
| 0.new migrants | 1 (reference) | 1 (reference) | 1 (reference) | 1 (reference) |
| 1.established migrants | 1.42 (0.82–2.47) | 1.38 (0.82–2.33) | 1.66 (0.97–2.85) | 0.90 (0.45–1.81) |
| 2.non-migrants | 2.71 (1.23–5.97) | 2.43 (1.25–4.72) | 2.93(1.38–6.24) | 0.85 (0.37–1.92) |
| Age (0=<18yrs,1=18yrs & above) | 4.70 (2.87–7.68) | 2.68 (1.58–4.55) | 4.03 (2.48–6.53) | 5.30 (2.28–12.33) |
| Schooling status (0=out of school,1=in school) | 0.33 (0.15–0.76) | 0.77 (0.33–1.79) | 0.34 (0.15–0.78) | 0.12 (0.01–1.04) |
| Parenthood status (0=Orphan,1=non-orphan) | 1.04 (0.65–1.67) | 0.64 (0.04–1.00) | 1.09 (0.69–1.73) | 0.62 (0.34–1.13) |
| Daily income in USD (0=<1USD,1=>1USD) | 1.21 (0.72–2.03) | 1.37 (0.81–2.30) | 1.05 (0.63–2.01) | 2.03 (0.95–4.34) |
| Religion (0=Non-Christian,1=Christian) | 1.07 (0.59–1.95) | 0.86 (0.49–1.52) | 1.14 (0.63–2.03) | 0.30 (0.15–0.62) |
| Marital status (0=Not married,1=married) | 1.61 (0.75–3.45) | 1.83 (0.93–3.59) | 1.77 (0.84–3.73) | 0.79 (0.32–1.97) |
| Received SRH education in past 6 months(0=no,1=yes) | 2.71 (1.64–4.46) | 2.10 (1.33–3.33) | 3.05 (1.88–4.95) | 1.41 (0.76–2.60) |
| Knows of a place to go to for FP (0=no,1=yes) | 3.23 (2.0–5.24) | 2.60 (1.65–4.11) | 2.77 (1.74–4.40) | 10.44 (4.87–22.38) |
| *Constant* | *0.21 (0.09–0.46)* | *0.99 (0.04–0.23)* | *0.18 (0.08–0.37)* | *0.03 (0.01–0.09)* |
| *Pseudo r-squared* | *0.2441* | *0.1573* | *0.2285* | *0.2915* |
